# The Examination of the Musculoskeletal System Based Only on the Evaluation of Pelvic-Hip Complex Muscle and Trunk Flexibility May Lead to Failure to Screen Children for Generalized Joint Hypermobility

**DOI:** 10.1371/journal.pone.0121360

**Published:** 2015-03-18

**Authors:** Dariusz Czaprowski, Agnieszka Kędra, Paulina Pawłowska, Aleksandra Kolwicz-Gańko, Justyna Leszczewska, Marcin Tyrakowski

**Affiliations:** 1 Department of Rehabilitation, University of Physical Education, Marymoncka 34, 00-968 Warsaw, Poland; 2 Department of Physiotherapy, Józef Rusiecki University College in Olsztyn, Bydgoska 33, 10-243 Olsztyn, Poland; 3 Department of Posture Correction and Compensation, Faculty of Physical Education and Sport in Biała Podlaska, Józef Piłsudski University of Physical Education in Warsaw, Akademicka 2, 21-500 Biała Podlaska, Poland; 4 Department of Physical Education, University of Physical Education, Marymoncka 34, 00-968 Warsaw, Poland; 5 Department of Orthopedics, Pediatric Orthopedics and Traumatology, The Centre of Postgraduate Medical Education in Warsaw, Konarskiego 13, 05-400 Otwock, Poland; The University of Queensland, AUSTRALIA

## Abstract

**Objective:**

The aim of the study was to evaluate whether the clinical assessment of the pelvic-hip complex muscle and trunk flexibility is sufficient for diagnosing generalized joint hypermobility (GJH).

**Design:**

A cross-sectional study.

**Setting:**

Center of Body Posture in Olsztyn, North East Poland.

**Participants:**

The study included 136 females and 113 males aged 10–13 years.

**Main outcome measures:**

In order to assess muscle flexibility, the straight leg raise (SLR) test (for hamstring) and modified Thomas test for one- (O-JHF) and two-joint (T-JHF) hip flexors were performed. To evaluate trunk flexibility the fingertip-to-floor (FTF) and lateral trunk flexion (LTF) tests were used. The GJH occurrence was assessed with the use of nine-point Beighton scale (threshold value ≥5 points for females, ≥4 for males). The analysis was carried out separately for females and males.

**Results:**

There were no significant differences between females with versus without GJH, and males with versus without GJH regarding SLR (p = 0.86, p = 0.19 for females and males, respectively), O-JHF (p = 0.89, p = 0.35 for females and males, respectively), T-JHF (p = 0.77, p = 0.4 for females and males, respectively), FTF (p = 0.19, p = 0.84 for females and males, respectively) and LTF (p = 0.58, p = 0.35 for females and males, respectively) tests results.

**Conclusions:**

Clinical examination of the pelvic-hip complex muscles and trunk flexibility by use of SLR, O-JHF, T-JHF, FTF and LTF revealed to be insufficient in diagnosing GJH in children aged 10–13 years. Thus, the Beighton scale should be considered a standard element of physiotherapeutic examination of the musculoskeletal system in children and youth.

## Introduction

Generalized joint hypermobility (GJH) is defined as an increased mobility of small and large joints in the particular age, gender and race, when systemic diseases are excluded [1]. GJH coexists with: spinal and peripheral joint pain and postural disorders, e.g. functional scoliosis and plano-valgus feet [2]. Generalized joint hypermobility is also more frequent in children and adolescents with idiopathic scoliosis than in a control group [3]. GJH may lead to joint damage, resulting in joint instability, subluxation and displacement [4]. The prevalence of GJH is reported to be 7% up to 65% and it appears more frequently in younger children, females as well as Asians and Africans [2,4–9]. Various clinical tests have been used to assess GJH with the 9-point Beighton scale being one of the most commonly applied [2,10].

Improvement in stabilization, balance and coordination are suggested to be the main goals of exercises in subjects with increased joint mobility [4,11,12]. Stretching exercises are not recommended for these individuals since they may be harmful and exert negative influence on hypermobile joints [12,13].

Physiotherapy programs, physical education classes as well as sport activities are commonly aimed at increasing flexibility of the pelvic-hip complex muscles [14–22]. Stretching exercises might be used as a warm-up or as a primary aim of the physiotherapy session [14,17,21]. These exercises are usually applied on the basis of the results of tests assessing muscle and trunk flexibility [14–17,19,20,22]. The most common tests used for this are straight leg raise test, modified Thomas test, fingertip-to-floor distance test and lateral trunk flexion test [23–26].

However, before stretching exercises the presence of subjects with GJH in the exercised group is likely to be disregarded [19,20,27]. The reasons for that may be: (1) the paucity of exact guidelines regarding stretching exercises when children with GJH in the exercised group exist, (2) the tests assessing muscle flexibility only, without GJH-specific tests are used in planning the exercises; and (3) insufficient knowledge of physical therapists concerning the principles of managing individuals with increased joint mobility [14,17,21,23,28–30].

To our knowledge there has not been any research verifying whether the use of tests assessing pelvic-hip complex muscle and trunk flexibility is sufficient for diagnosing GJH and at the same time for optimal planning of the exercises.

Therefore, the main aim of the study was to assess whether the straight leg raise, modified Thomas, fingertip-to-floor and lateral trunk flexion tests make it possible to diagnose GJH or whether it is necessary to supplement the clinical evaluation of the musculoskeletal system with a GJH specific test, i.e. the Beighton scale. In order to achieve this aim, the pelvic-hip complex muscle and trunk flexibility in females and males with and without GJH aged 10–13 years was compared.

## Materials and Methods

### Participants

The subjects were selected prospectively during meetings organized for children and their parents. The meetings were held in 6 randomly selected primary schools. The schools were selected by use of simple randomization by employing computer-generated number of the school. All students from grades 4, 5 and 6 (aged 10–13 years) and their parents were invited for the meetings. 350 Caucasian children with parents participated in the meetings. Detailed information regarding aims, schedule and protocol of the study as well as inclusion criteria were presented. The inclusion criteria were: (1) written consent of a parent or a guardian of the participant; (2) age of the participant between 10 and 13 years; (3) no trauma or pain episode within 3 months prior to the study; (4) no known orthopaedic, neurological, rheumatological or genetic disorders.

Two hundred and forty-nine subjects (136 females and 113 males) met the inclusion criteria.

Height and weight of each participant were measured by use of stadiometer and medical scale (TRYB WAG, Bydgoszcz, Poland) with accuracy of 1.0cm and 0.1kg, respectively. Next, the body mass index (BMI) was calculated. Detailed characteristics of the study group are presented in Table 1.

**Table 1:** Parameters of the study group (females, n = 136 and males, n = 113).

Variable	Mean	SD	Median	QR	Minimum	Maximum
	F/M	F/M	F/M	F/M	F/M	F/M
Age (years)	11.7/11.8	0.7/0.8	12.0/12.0	1.0/1.0	10.0/10.0	13.0/13.0
Height (cm)	151.1/151.2	8.0/8.8	151.0/150.0	11.0/13.0	130.0/135.0	169.0/174.0
Weight (kg)	43.9/44.9	10.8/13.6	42.0/41.3	14.0/16.1	21.0/24.0	78.0/92.0
BMI (kg m-2)	19.1/19.4	3.8/4.3	18.4/18.1	4.4/5.3	11.0/11.9	29.9/35.1

Abbreviations: F—females; M—males; SD—standard deviation; QR—Quartile Range.

Prior to the study, a written parents’ consent was obtained. The Józef Rusiecki University College Ethical Commission approved the study.

**Fig 1:**
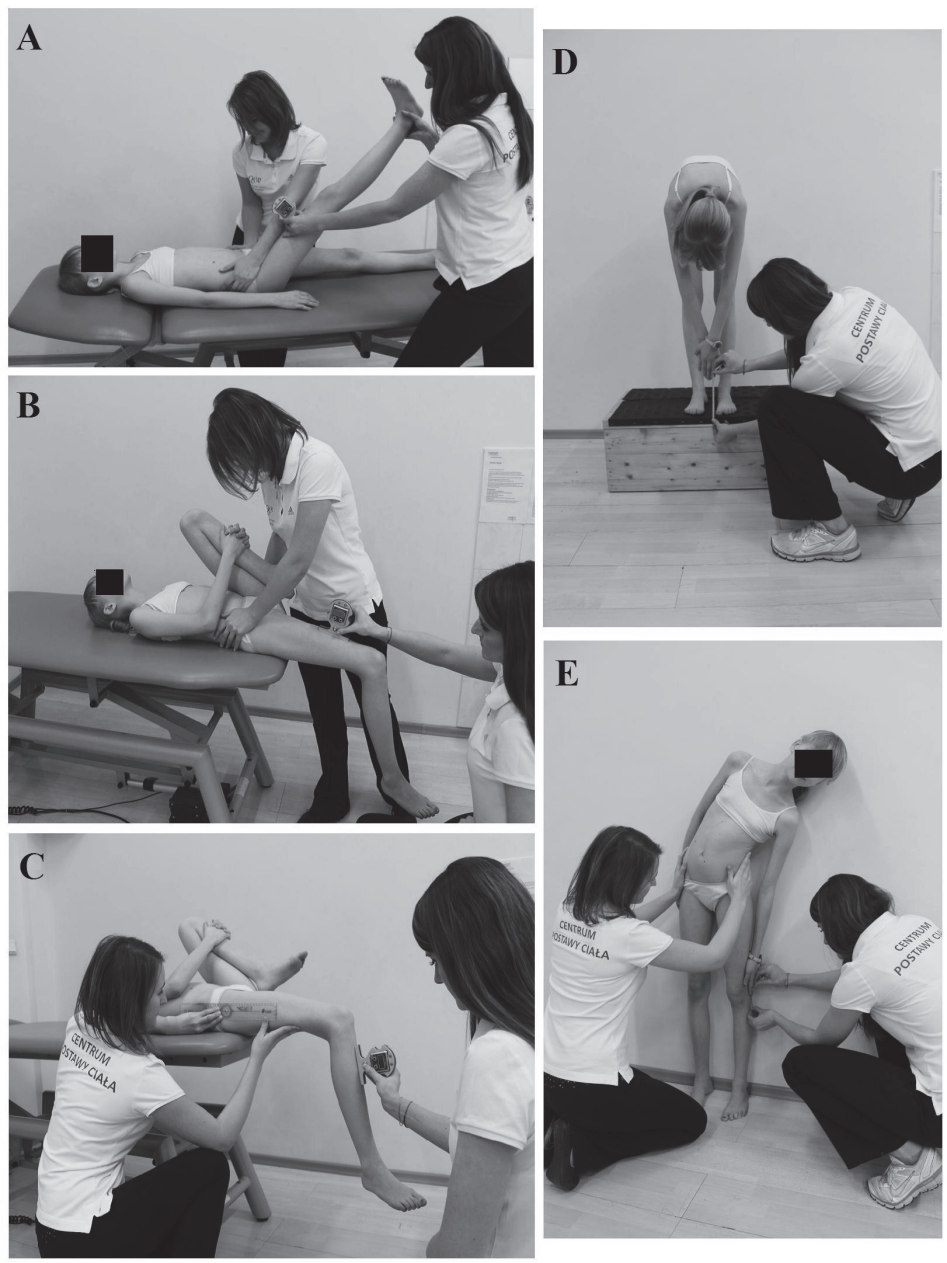
The tests used in the study: A—straight leg raise test, B—modified Thomas test for one-joint hip flexors, C—modified Thomas test for two-joint hip flexors, D—fingertip-to-floor test, E—lateral trunk flexion test.

The legal guardian of the subject presented in Fig. 1 has given written informed consent (as outlined in PLOS consent form) for the publication of her photographs. The other two persons are the co-authors of the study and have also provided a written informed consent for publication.

### Assessment of the pelvic-hip complex muscle and trunk flexibility

All of the subjects underwent primary clinical evaluation of hamstrings and one- and two-joint hip flexors flexibility as well as fingertip-to-floor and lateral trunk flexion tests.

Examination was performed simultaneously by two researchers (physiotherapists with 5 years of practice). The first researcher (R1) conducted all of the tests and measurements by use of AMI Digital Inclinometer (OPIW, Poland) with accuracy of 1.0° and a flexible tape measure with accuracy of 0.5cm. The second researcher (R2) controlled visually and with palpation the subjects’ position during examination, namely stable position of the pelvis and/or flat position of the lumbar spine.

All of the measurements were performed three times for each subject, and mean values were taken for further analysis.

#### Hamstring flexibility assessment

Hamstring flexibility was evaluated by use of the straight leg raise test (SLR) [23,24]. The test was performed with the subject in supine position with lower extremities extended in the knee joints and feet relaxed. R2 evaluated visually position of the trunk and stabilized the subject’s pelvis manually, while R1 started to raise one of the subject’s lower extremities by passively flexing the hip with the knee extended. The angle of flexion in the hip joint at which the subject felt resistance in the posterior thigh or flexed the knee was the end result of the test and was measured using the inclinometer placed on the anterior aspect of the thigh (10cm proximally to the knee joint). Before the test, the inclinometer was reset in a horizontal position (Fig. 1A). This procedure was repeated for the contralateral lower extremity.

#### Hip flexors assessment

Modified Thomas test was used to evaluate the flexibility of one- (O-JHF) and two-joint hip flexors (T-JHF) [15,23].

In order to evaluate O-JHF the subject was positioned in a supine position on the table with the pelvis close to the edge of the table. The contralateral lower extremity was flexed in the knee and hip joint and held by the subject with the thigh close to the chest. R2 stabilized the subject’s pelvis and observed proper position of the lumbar spine (lying flat on the table). The examined lower extremity was held in a relaxed position. R1 measured the angle of flexion/extension in the hip by placing the inclinometer (previously reset in a horizontal position) on the anterior aspect of the thigh of the examined extremity (10cm proximally to the knee joint). When the hip joint was in flexion, negative values of the angle were noted (marked with a ‘minus’). When the hip was in extension, positive values of the angle were noted (Fig. 1B).

In order to assess T-JHF the subject was positioned in a supine position on the table with the pelvis close to the edge of the table. The contralateral lower extremity was flexed in the knee and hip joint and held by the subject with the thigh close to the chest. R2 observed proper position of the lumbar spine (lying flat on the table) and controlled the neutral (parallel to the table) position of the thigh of the examined lower extremity (Fig. 1C). This position was controlled by use of a goniometer placed with its fulcrum over the greater trochanter, the immobile arm parallel to the table and the mobile arm along the axis of the thigh. The knee joint of the examined lower extremity was relaxed and the angle of flexion in the knee was measured by R1 using the inclinometer placed just distally to the tibial tuberosity.

#### Fingertip-to-floor test (FTF)

Fingertip-to-floor test was performed with the subject standing barefoot on a measurement box with known height of 30cm [25]. The subject bent forward (with the knee joints extended) and the distance from the fingertips to the top of the box was measured by use of a flexible tape measure and noted in cm with accuracy of 0.5cm. When the subject could not reach the top of the measurement box negative values were noted (marked with ‘minus’). Positive values were obtained when the subject was able to touch the box below its superior surface (Fig. 1D).

#### Lateral trunk flexion (LTF)

Evaluation of LTF was performed in a standing position with feet positioned hip-width apart and the posterior aspect of the trunk maintaining contact with the wall (Fig. 1E) [23,26]. The upper extremities were freely suspended at both sides of the body. R2 stabilized the subject’s pelvis in neutral position. Subsequently the child was requested to flex the trunk laterally. The subject was asked to touch the lowest point on his lower extremity with extended fingers of the hand. The distance between the subject’s fingertip and the tip of the fibular head was measured by use of flexible tape measure with 0.5cm accuracy. Negative values of LTF were noted when the child reached the point proximal to the fibular head. When the point distal to the fibular head was reached, positive values of LTF were noted.

### Pilot reliability study

Prior to the study, reproducibility of all the tests used for the evaluation of the pelvic-hip complex muscle and trunk flexibility was assessed on 10 children (7 females and 3 males) randomly selected from the study group. All the tests were performed twice with 1 week apart by the same two researchers (R1 and R2). The methodology of each test was as described above. Intraobserver reproducibility of all the tests was quantified by intraclass correlation coefficient (ICC) and median error for a single measurement (SEM) [31].

### Assessment of generalized joint hypermobility

A third researcher who did not participate in the flexibility testing, and was blinded to these results, evaluated GJH in all of the subjects using the 9-point Beighton scale [4,10,32]. The scale includes the evaluation of: (1) extension of the metacarpophalangeal joint in the 5th finger above 90 degrees, (2) abduction of the thumb to the forearm, (3) elbow hyperextension above 10 degrees, (4) knee hyperextension above 10 degrees, and (5) placing flat hands on the floor with straight legs. Each hypermobile joint is given 1 point [4,10]. GJH was diagnosed when 5 points or more were noted for females and 4 points or more for males, respectively [33]. Thus, the study group could be divided into subgroups: subjects with GJH (GJH group) and subjects without GJH (NH group).

### Statistical analysis

Statistical analysis was performed with Statistica 7.1 (StatSoft, Poland). Descriptive statistics (means and standard deviations, median and quartile range, and minimum and maximum values) were calculated for all females and males and separately for females and males with and without GJH. Normal distribution of the data was analyzed by use of Shapiro-Wilk test. Unpaired t-test and Mann-Whitney U test were used to assess the differences in means of particular parameters. Statistical significance was set at P<0.05 with 95% confidence interval. Power of the statistical tests was set at the level of 0.8 and the least significant difference (LSD) detected was calculated for each particular comparison.

Due to the fact that in the research different criteria for diagnosing GJH for females and males were applied and due to differences in pelvic-hip muscle and trunk flexibility between females and males [23,34,35], statistical analysis was conducted separately for both genders.

The ICC value of less than 0.40 indicated poor reproducibility/reliability, 0.40–0.75 indicated fair to good reproducibility/reliability, and values greater than 0.75 reflected excellent reproducibility/reliability [36].

## Results

### Pilot reliability study

All of the tests used in the study revealed good to excellent reproducibility with the following values of ICC and SEM: SLR: 0.95 and 1.0°; O-JHF: 0.75 and 1.4°; T-JHF: 0.95 and 1.2°; FTF: 0.99 and 0.5cm; and LTF: 0.9 and 1.0cm.

### Assessment of the pelvic-hip complex muscles and trunk flexibility

The results of SLR, O-JHF, T-JHF, FTF and LTF tests for females and males are presented in Tables 2 and 3, respectively.

**Table 2:** The results of the pelvic-hip complex muscles and trunk flexibility tests in females (n = 136).

	Mean	SD	Median	QR	Minimum	Maximum
SLR right (°)	57.0	8.2	57.0	10.0	34.0	78.0
SLR left (°)	57.5	8.7	57.0	12.0	34.0	78.0
O-JHF right (°)	13.0	8.7	15.0	7.0	-22.0	30.0
O-JHF left (°)	14.0	8.1	13.0	8.0	-21.0	32.0
T-JHF right (°)	84.1	4.8	84.0	6.0	69.0	90.0
T-JHF left (°)	84.4	4.8	84.0	6.0	67.0	90.0
FTF (cm)	-2.9	9.0	0.0	8.0	-34.0	17.0
LTF right (cm)	0.0	4.1	0.0	6.0	-12.0	9.0
LTF left (cm)	-0.5	4.0	0.0	5.0	-14.0	9.0

Abbreviations: SD—standard deviation; QR—Quartile Range; SLR—straight leg raise test; O-JHF—one-joint hip flexors test; T-JHF—two-joint hip flexors test; FTF—fingertip-to-floor test; LTF—lateral trunk flexion test.

**Table 3:** The results of the pelvic-hip complex muscles and trunk flexibility tests in males (n = 113).

	Mean	SD	Median	QR	Minimum	Maximum
SLR right (°)	50.5	9.4	51.0	12.0	24.0	75.0
SLR left (°)	51.0	9.0	51.0	11.0	26.0	79.0
O-JHF right (°)	13.2	8.6	14.0	7.0	−13.0	33.0
O-JHF left (°)	13.8	7.9	15.0	7.0	−15.0	28.0
T-JHF right (°)	84.5	4.9	85.0	6.0	66.0	93.0
T-JHF left (°)	84.1	6.2	85.0	8.0	62.0	98.0
FTF (cm)	−7.4	8.9	−6.0	14.0	−25.0	16.0
LTF right (cm)	−0.5	3.4	0.0	5.0	−10.0	8.0
LTF left (cm)	−0.7	3.7	0.0	5.0	−11.0	10.0

Abbreviations: SD—standard deviation; QR—Quartile Range; SLR—straight leg raise test; O-JHF—one-joint hip flexors test; T-JHF—two-joint hip flexors test; FTF—fingertip-to-floor test; LTF—lateral trunk flexion test.

### Assessment of generalized joint hypermobility

Eighteen females (13.2%; 18/136) met the diagnostic criteria of GJH (GJH females group) with an average 9-point Beighton scale score of 6.6 (±1.3). The remaining 118 females with no GJH (NH females group) had an average 9-point Beighton scale score of 1.4 (±1.5). Nineteen males (16.8%; 19/113) met the diagnostic criteria of GJH (GJH males group) with an average 9-point Beighton scale score of 5.6 (±1.7). Ninety-four males without GJH (NH males group) had an average 9-point Beighton scale score of 0.7 (±1.0).

Age, height, weight and BMI were not significantly different between the NH and GJH groups, both for females and males (Table 4 and Table 5)

**Table 4:** Comparison of age, height, weight, BMI as well as hamstring, one- and two-joint hip flexors flexibility, fingertip-to-floor and lateral trunk flexion test results between non-hypermobile females (NH females group) and females with generalized joint hypermobility (GJH females group).

	NH females group	GJH females group	P	LSD
	n = 118	n = 18		
Age (years)	12.0 (1.0)	11.0 (1.0)	0.08*	0.35
Height (cm)	151.0 (7.9)	151.9 (9.0)	0.65†	4.0
Weight (kg)	41.3 (14.0)	46.5 (16.5)	0.19*	5.4
BMI (kg m-2)	18.2 (4.3)	19.7 (6.7)	0.24*	1.9
SLR (°)	57.0 (8.3)	57.3 (7.7)	0.86*	4.1
O-JHF (°)	15.0 (8.0)	15.0 (6.0)	0.89*	4.4
T-JHF (°)	84.0 (6.0)	84.0 (7.0)	0.77*	2.4
FTF (cm)	0.0 (9.0)	0.0 (4.0)	0.19*	4.5
LTF (cm)	0.0 (6.0)	0.5 (7.0)	0.58*	2.1

Abbreviations: SLR—straight leg raise test; O-JHF—one-joint hip flexors test; T-JHF—two-joint hip flexors test; FTF—fingertip-to-floor test; LTF—lateral trunk flexion test.

† Values are in mean (SD) and by independent t-test;

* Values are in median (QR) and by Mann-Whitney U test. Statistically significant differences are in bold;

LSD—least significant difference detected by statistical analysis with 0.8 power of the test.

**Table 5:** Comparison of age, height, weight, BMI as well as hamstring, one- and two-joint hip flexors flexibility, fingertip-to-floor and lateral trunk flexion tests results between non-hypermobile males (NH males group) and males with generalized joint hypermobility (GJH males group).

	NH males group	GJH males group	P	LSD
	n = 94	n = 19		
Age (years)	11.8 (0.8)	11.8 (0.7)	0.89†	0.4
Height (m)	150.7 (8.9)	153.5 (8.1)	0.19†	4.4
Weight (kg)	44.3 (13.4)	48.0 (14.2)	0.18†	6.7
BMI (kg m-2)	19.2 (4.3)	20.1 (4.6)	0.42†	2.1
SLR (°)	50.0 (9.3)	53.1 (9.3)	0.19*	4.6
O-JHF (°)	14.0 (6.0)	15.0 (7.0)	0.35*	4.3
T-JHF (°)	85.0 (7.0)	86.0 (5.0)	0.40*	2.5
FTF (cm)	−6.0 (14.0)	−7.0 (13.0)	0.84*	4.5
LTF (cm)	−0.7 (3.5)	0.2 (4.1)	0.35*	1.8

Abbreviations: SLR—straight leg raise test; O-JHF—one-joint hip flexors test; T-JHF—two-joint hip flexors test; FTF—fingertip-to-floor test; LTF—lateral trunk flexion test.

† Values are in mean (SD) and by independent t-test;

* Values are in median (QR) and by Mann-Whitney U test. Statistically significant differences are in bold;

LSD—least significant difference detected by statistical analysis with 0.8 power of the test.

### Comparison between subjects with and without GJH

There were no statistically significant differences between right and left side of the body for all the tests performed (SLR, P = 0.62, and P = 0.69; O-JHF, P = 0.29, and P = 0.55; T-JHF, P = 0.65, and P = 0.62; LTF, P = 0.31, and P = 0.76, for females and males, respectively). Thus, the results obtained for the right side served as references and were used in the further analysis.

No statistically significant difference in SLR, O-JHF, T-JHF, FTF and LTF between GJH females versus NH females as well as GJH males versus NH males were found (Table 4 and Table 5).

## Discussion

We present a study aimed at verifying if the clinical examination of the pelvic-hip complex muscle and trunk flexibility is sufficient for diagnosing GJH in children aged 10–13 years. To the authors’ knowledge such an analysis has never been performed.

We found no significant differences in SLR, O-JHF, T-JHF, FTF, and LTF tests between subjects with and without GJH for both females and males. Therefore, the results of the presented study indicate that the SLR, O-JHF, T-JHF, FTF and LTF tests cannot be used as indicators of GJH. Lack of difference in FTF test results between children with and without GJH is of note, considering FTF is a part of the of 9-point Beighton scale. A positive result of this test was observed only in 8 females and in 3 males with GJH (22% and 16% all of females and males with GJH, respectively). We suspect that this finding may be due to the influence of both, mobility of the spine and hamstring flexibility (that was low in GJH groups with the mean range of hip flexion 57.3°±7.7 and 53.1°±9.3 for females and males with GJH, respectively) on FTF test result.

Greenwood et al. observed an increased activity of semitendinosus in subjects with GJH. According to these authors it might be a compensatory mechanism for pelvic instability [37]. In our research no differences between children with and without GJH were noted in the analysed tests despite the fact that increased laxity of the connective tissue (ligaments, articular capsules) is characteristic of GJH. Taking into account the suggestion put forward by Greenwood et al. [37], perhaps the noted lack of increased range of motion in children with GJH may result from an increased compensatory muscle activity. However, it is necessary to carry out further research (with the use of, inter alia, electromyography) which will make it possible to fully assess the state of musculoskeletal system in individuals with generalized joint hypermobility.

Our findings correspond to the data presented by Smits-Engelsman et al. (2011) [32]. Although they observed slightly higher range of flexion and extension in the hip joints in children with GJH, the mean difference in hip flexion and extension between results obtained by children with and without GJH were small (3.2° and 1.6° for hip flexion and extension, respectively) and similar to the least significant differences noticed in our study (4.1° and 4.6° for hip flexion and 4.6° and 4.3° for hip extension in females and males, respectively).

However, it is worth noting that in their research Smits-Engelsman et al. (2011) [32] assessed children without any relation to gender differences. Taking into account different criteria for GJH diagnosis suggested in the references [33] and differences in pelvic-hip complex muscle and trunk flexibility between females and males [34,35] our study has provided gender-specific data, which, in our opinion, constitutes a significant value of the presented research.

### Clinical relevance

Children and adolescents participating in physiotherapy programs, school physical education classes as well as amateur and professional sport activities commonly perform exercises aimed at increasing flexibility of the trunk and the pelvic-hip complex muscles [15,16,18–20,22]. These exercises might be used as a warm-up or as a primary aim of the lesson or workout. The main reason for the use of stretching exercises is the result of tests aimed at assessing muscles and trunk flexibility [14,17,21,23,28,29].

An analysis of the literature as well as authors’ observations indicate that children are not routinely screened with respect to GJH occurrence prior to prescription of exercises, physical activity or therapy [14,16,17,19–23,27–29]. This is consistent with observations made by Russek (1999) who claimed that individuals with joint hypermobility may be disregarded in standard physical examination. Thus, subjects with joint hypermobility may be under- or misdiagnosed [13]. However, Russek (1999) [13] made his observations for individuals with hypermobility syndrome (HS) that is clinically symptomatic in contrast to asymptomatic GJH [4]. However, the borderline between constitutional GJH and pathological skin and joint laxity is not always easily defined [3]. Nevertheless, clinical examination still remains the standard method of evaluating both GJH and pathological soft tissue laxity in everyday practice.

Muscle strengthening as well as proprioceptive and stability training are recommended in management of children with HS [4,9,11–13]. On the other hand, stretching exercises should not be performed due to their potential negative influence on hypermobile joints [12]. This is supported by the results of the study conducted by Howell (1984), who observed increased incidence of back pain in individuals with excessive spinal mobility who participated in a stretching program [38]. However, recommendations regarding specific exercises for patients with joint hypermobility continue to be founded on expert opinion, rather than evidence based physiotherapy [13].

The need to differentiate exercises for individuals with asymptomatic joint hypermobility is reflected in the observations made by Greenwood et al. (2011).

These authors observed the differences in muscle activation patterns between pain-free hypermobile and control subjects, specifically involving muscles surrounding the pelvis. According to the authors, lower activity of the erector spinae and greater activity of the semitendinosus as well as higher levels of rectus femoris-semitendinosus co-contraction might increase the risk of certain clinical conditions for asymptomatic hypermobile individuals [37]. Specificity in exercise prescription for asymptomatic individuals with GJH may therefore be prudent.

Taking into account the above mentioned recommendations, our findings are clinically relevant, because they indicate that evaluation of the musculoskeletal system based only on the selected muscle tests and basic tests assessing trunk flexibility without joint hypermobility specific tests may be insufficient to identify children with GJH. Consequently, an evaluation limited to such tests may lead to non-optimal planning of exercise prescription. Thus, we recommend to implement the Beighton scale into the standard basic clinical examination performed by physiotherapists or physical education teachers.

It is also worth highlighting that the tests assessing pelvic-hip complex muscle and trunk flexibility which were applied in this research are widely used in clinical practice and scientific research verifying various techniques aimed at increasing muscle flexibility. Therefore, in our opinion, the results of the presented study may be useful while planning the exercises and in further research assessing the effectiveness of stretching exercises.

### Limitations and future recommendations

The majority of the papers describing increased joint mobility concerns the joint hypermobility syndrome, and there is a paucity of literature concerning GJH [4,7,11–13]. Generalized joint hypermobility and HS have a similar clinical presentation with one key difference between them—arthralgia develops only in HS [4]. Nevertheless, we suggest that therapeutic recommendations for HS (avoiding joint stretching exercises) may be used in individuals with GJH. However, further studies verifying this assumption are needed.

## Conclusions

Clinical examination of the pelvic-hip complex muscle and trunk flexibility by use of SLR, O-JHF, T-JHF, FTF and LTF tests was insufficient in diagnosing GJH in children aged 10–13 years. Thus, the Beighton scale should be considered a standard element of physiotherapeutic examination of the musculoskeletal system in children and youth.
